# Harsh parenting and child conduct and emotional problems: parent- and child-effects in the 2004 Pelotas Birth Cohort

**DOI:** 10.1007/s00787-021-01759-w

**Published:** 2021-03-18

**Authors:** Andreas Bauer, Graeme Fairchild, Sarah L. Halligan, Gemma Hammerton, Joseph Murray, Ina S. Santos, Tiago N. Munhoz, Aluísio J. D. Barros, Fernando C. Barros, Alicia Matijasevich

**Affiliations:** 1 Department of Psychology, 10 West, University of Bath, Claverton Down, Bath, BA2 7AY UK; 2 Population Health Sciences, Bristol Medical School, University of Bristol, Bristol, UK; 3 Postgraduate Program in Epidemiology, Federal University of Pelotas, Pelotas, Brazil; 4 Postgraduate Program in Pediatrics and Child Health, Pontifical Catholic University of Rio Grande Do Sul, Porto Alegre, Brazil; 5 Faculty of Psychology, Federal University of Pelotas, Pelotas, Brazil; 6 Postgraduate Program in Health and Behaviour, Catholic University of Pelotas, Pelotas, Brazil; 7 Department of Preventive Medicine, Faculty of Medicine FMUSP, University of São Paulo, São Paulo, Brazil

**Keywords:** Harsh parenting, Child abuse, Conduct problems, Emotional problems, Cross-lagged panel design, Transactional model

## Abstract

In high-income countries, links between harsh and abusive parenting and child conduct and emotional problems are well-documented. However, less is known about these relationships in low- and middle-income countries, where harsh parenting may be more widely accepted and higher rates of conduct or emotional problems may exist which could influence the strength of these associations. We sought to investigate these relationships in a large population-based, prospective longitudinal study from Brazil, which also allowed us to test for sex differences. Using data from the 2004 Pelotas Birth Cohort Study (*N* = 4231) at ages 6 and 11 years, we applied cross-lagged path analysis to examine the relationships between harsh parenting (Conflict Tactics Scale Parent–Child version), and child conduct and emotional problems (Strengths and Difficulties Questionnaire). We found reciprocal relationships between harsh parenting and child conduct problems, with harsh parenting at age 6 predicting child conduct problems at age 11, and vice versa, even after adjusting for initial levels of conduct problems and harsh parenting, respectively. For child emotional problems, only unidirectional effects were found, with harsh parenting at age 6 predicting child emotional problems at age 11, after adjusting for initial levels of emotional problems, but not vice versa. No significant sex differences were observed in these relationships. These observations based on a middle-income country birth cohort highlight the potential universality of detrimental effects of harsh parenting on child conduct and emotional problems and affirm the importance of addressing parent- and child-effects in preventive and treatment interventions, especially those targeting conduct problems.

**Supplementary Information:**

The online version contains supplementary material available at 10.1007/s00787-021-01759-w.

## Introduction

For decades, the role of parenting behaviors in the development of child psychopathology has been a major focus of research. Early *parent-effect* models, which proposed a unidirectional relationship from parenting to child outcomes [1], evolved to take into account *child-effects*, in which child characteristics and behaviors modify parental behaviors [2]. Thus, the coercive processes model proposes that the parents’ failure to maintain child compliance in their early interactions initiates a continuing cycle of dysfunctional exchanges. More precisely, ineffective parental demands in response to a child’s problem behavior (e.g., aggression or anger outbursts) are followed by the child’s refusal to comply, which, in turn, elicits further ineffective parenting (e.g., withdrawal) [3, 4]. Consequently, over time, the child’s aggressive behavior increases, and the parents’ capacity to regulate the child’s problematic behavior decreases.

Repetti et al.’s [5] model places a major emphasis on such neglectful and harsh family environments, which are proposed to result in emotional dysregulation in children, which, in turn, is thought to be implicated in the development of both externalizing and internalizing psychopathology. With respect to the latter, internalizing symptoms in children and adolescents are particularly linked to coping strategies involving disengagements, such as emotion suppression, avoidance, and denial [6]. Thus, unlike children showing externalizing problems who become ensnared in coercive exchanges with parents, siblings, and peers [4], those with internalizing symptoms may prevent or disrupt these vicious cycles by withdrawing from the hostile situation.

To date, two meta-analyses have examined the relationships between harsh parenting and child externalizing and internalizing problems, respectively [7, 8]. In the first, Pinquart examined whether later externalizing symptoms are predicted by harsh parenting at earlier stages of development, after adjusting for initial levels of externalizing symptoms, and vice versa [7]. Consistent with transactional models of developmental psychopathology, Pinquart found bidirectional effects between harsh parenting and child externalizing symptoms, i.e., harsh parenting led to higher rates of externalizing symptoms in the child, while externalizing problems in the child elicited more harsh parenting over time [7]. In contrast, in the second meta-analysis of cross-lagged associations between harsh parenting and child internalizing problems, Pinquart [8] found only a unidirectional effect, whereby harsh parenting predicted internalizing problems, but *not* vice versa. In sum, harsh parenting appears to be reciprocally related to child externalizing symptoms, whereas only a unidirectional relationship from harsh parenting to child internalizing symptoms has been observed.

However, it should be noted that the vast majority (96%) of the studies included in the meta-analysis on cross-lagged associations between harsh parenting and child externalizing symptoms were from high-income countries (HICs) [7] (the relevant information could not be extracted from the meta-analysis on internalizing symptoms [8]). Just four studies were conducted in low- and middle-income countries (LMICs), and only one of these was population-based [9]. This lack of evidence from LMICs is also reflected in a third meta-analysis by Pinquart and Kauser [10], in which country-level differences in cross-lagged associations between harsh parenting and child externalizing and internalizing problems could not be estimated due to the small number of studies from non-Western countries.

This gap in the evidence base is concerning, given that almost 90% of all children and adolescents worldwide live in LMICs [11]. Importantly, effects of harsh parenting on child externalizing and internalizing problems may depend partly on cultural norms. In particular, it is proposed that the effects may be attenuated in countries in which harsh punishment is more common and widely accepted [12, 13]. For example, Lansford et al. [12] found that corporal punishment led to increased levels of child externalizing and internalizing problems across low-, middle-, and high-income countries. However, effect sizes were smaller in countries in which corporal punishment was perceived as more normative [12]. Although the use of physical and verbal punishment is common worldwide, there is considerable between- and within-country variability [14], especially for more severe forms of harsh parental discipline [15]. Consequently, the effects of harsh parenting on child externalizing and internalizing problems may differ across countries, and it remains unclear if the findings obtained in HICs translate to LMICs.

To address these gaps in the literature, we examined the association between harsh, aggressive, or abusive parenting (hereafter referred to as *harsh parenting*), defined as physical and psychological aggression towards the child, and child externalizing and internalizing symptoms, in the 2004 Pelotas Birth Cohort study. This is a large population-based sample based in Brazil, a middle-income country with high levels of crime and violence, especially amongst adolescents [16, 17]. The main objectives of the present study were: (i) to test whether harsh parenting is associated with child conduct and emotional problems in a LMIC context; and (ii) to examine whether there are unidirectional or reciprocal relationships between harsh parenting and child conduct and emotional problems, using autoregressive path models to test for cross-lagged associations. In line with previous research [7, 8], we hypothesized that harsh parenting would be reciprocally related to child conduct problems, whereas only a unidirectional relationship would be observed between harsh parenting and child emotional problems. Given that examination of sex differences has been limited in previous research—even when considering HICs [7, 8]—we tested whether the effects of harsh parenting vary according to the sex of the child, and also whether the stability of child externalizing and internalizing symptoms differs by sex.

## Methods

### Participants

The 2004 Pelotas Birth Cohort is a population-based, prospective longitudinal study, investigating the impact of early life exposure to a wide range of risk factors on maternal and child health outcomes [18, 19]. Pelotas, Rio Grande do Sul (South Brazil) has a population of approximately 340,000 people, predominantly residing in urban areas (93%), with 98% of births occurring in hospitals. Out of the 4263 live births in 2004 identified through daily hospital visits, 4231 (99.2%; 51.9% boys) were included and mothers were interviewed within 24 h postpartum. Mother–child dyads were assessed again at ages 3 (99.2%), 12 (95.7%), 24 (93.5%), and 48 (92.0%) months, and when the child was 6 (90.2%) and 11 (86.6%) years of age. Data were collected during home visits up to when the children were aged 48 months, and in the study clinic at ages 6 and 11 years. The current sample was restricted to singletons (*N* = 4145; 52.0% boys). Further details about the cohort and the assessments undertaken can be found in Santos et al. [18, 19].

### Measures

#### Harsh parenting

Caregivers, the majority of whom were mothers at ages 6 (89.0%) and 11 (92.5%) years, were asked about harsh parenting strategies using the parent-to-child version of the Conflict Tactics Scale (CTSPC) [20]. The CTSPC comprises 22 items across three subscales measuring parental behaviors towards the child over the past 12 months related to non-violent discipline (4 items); psychological aggression (5 items); and physical assault, including corporal punishment (5 items), physical maltreatment (4 items), and severe physical maltreatment (4 items; not administered in this study). In line with two previously published meta-analyses [7, 8], we defined harsh parenting as comprising the sum scores of the psychological aggression (e.g., “Shouted, yelled, or screamed at him/her”), corporal punishment (e.g., “Spanked him/her on the bottom with our bare hands”), and physical maltreatment (e.g., “Slapped him/her on the face or head or ears”) subscales. All items were rated on a 3-point scale (0–2), from *never* to *once* and *more than once*, yielding overall scores ranging from 0 to 28. The Portuguese version of the CTSPC has been cross-culturally adapted and validated for use in Brazil [21, 22].

#### Conduct and emotional problems

Child conduct and emotional problems were measured at ages 6 and 11 years using the parent-rated conduct problems (e.g., “Often fights with other children or bullies them”) and emotional problems (e.g., “Many worries, often seems worried”) subscales of the Strengths and Difficulties Questionnaire (SDQ) [23]. We used individual subscales, as opposed to the externalizing problems subscale (which is an aggregate of the conduct problems and hyperactivity subscales), as research has indicated meaningful differences between these symptom clusters [24]. Each subscale comprises five items, which are rated on a 3-point scale (0–2), from *not true* to *somewhat true* and *certainly true*, yielding overall scores ranging from 0 to 10. The Portuguese version of the SDQ has been validated for use in Brazil [25, 26]. The scales showed modest internal reliability with Cronbach’s alphas of 0.59–0.65 and 0.52–0.59 for the conduct and emotional problems subscales, respectively.

#### Covariates

We included sociodemographic characteristics, prenatal environmental factors, and maternal psychopathology, which have been identified as risk factors for negative parenting and child conduct and emotional problems. Maternal depression was measured at 12 months after delivery using the self-reported Edinburgh Postnatal Depression Scale (EPDS) [27]. The 10 items are rated on a 4-point scale (0–3), with scores ranging from 0 to 30. The Portuguese version of the EPDS has been validated for use in Brazil [28]. Information on all other covariates was collected within 24 h postpartum by maternal self-report, unless otherwise stated. Mothers who smoked 1+ cigarettes daily during any trimester of pregnancy were classified as smoking during pregnancy. Any amount of alcohol intake during any trimester of pregnancy was considered as prenatal alcohol exposure. Maternal skin color was determined by the interviewer and, for the purposes of this study, classified as White versus Black/Mixed race. Mothers who were single, widowed, divorced, or who lived without a partner were classified as single mothers. Maternal education was coded as complete school years of formal education. Income was coded as the weekly family income in the month prior to the child’s birth.

### Analysis strategy

**Fig. 1:**
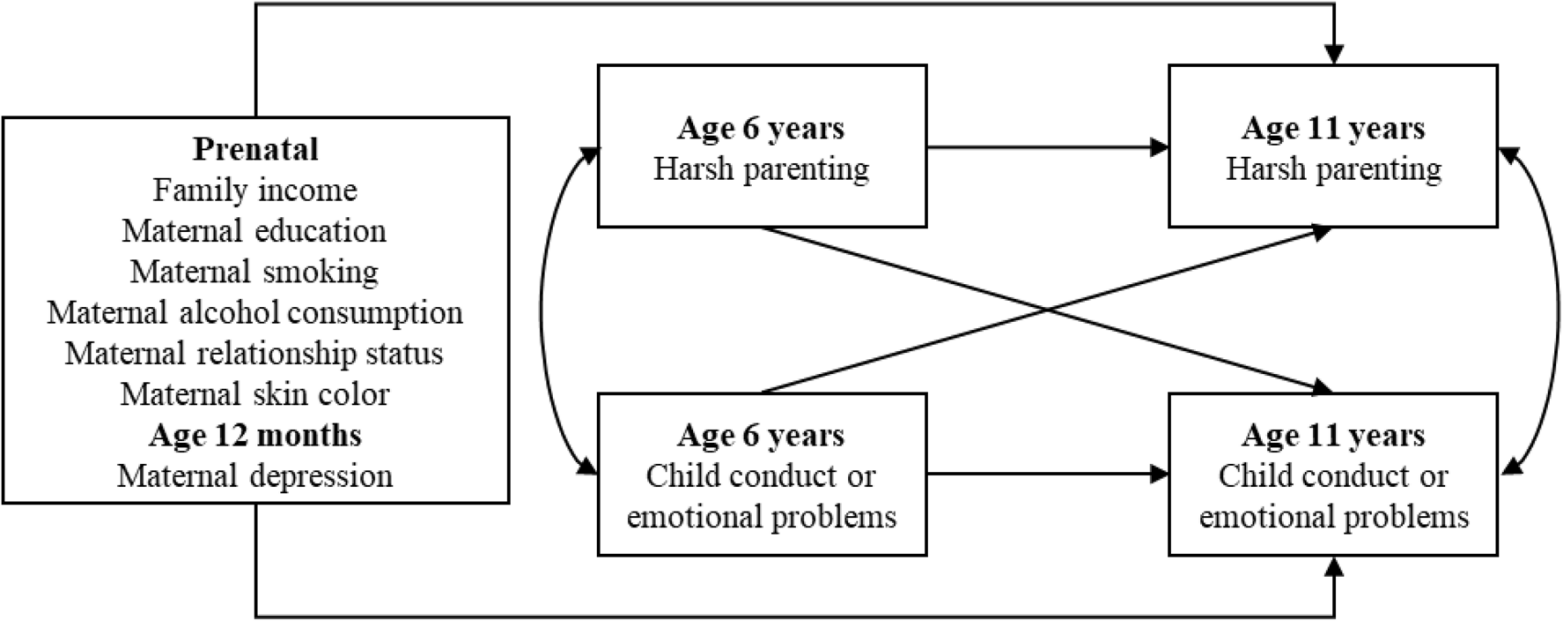
Schematic representation of an observed-variable autoregressive path model examining reciprocal interactions between harsh parenting and child conduct or emotional problems, after adjusting for covariates. Lines with single arrowheads represent hypothesised direct effects. Curved lines with two arrowheads represent correlations. Analyses were conducted separately for child conduct and emotional problems

We used observed-variable autoregressive path models to examine the reciprocal associations between harsh parenting and child conduct problems and emotional problems, respectively [29]. These models estimate the effect of one variable on another, temporally succeeding variable (i.e., cross-lagged effects), while also adjusting for the stability of each variable over time (i.e., autoregressive effects). Figure 1 presents a schematic diagram of reciprocal change between harsh parenting and child conduct and emotional problems, respectively.

The amount of missing data for the harsh parenting and child conduct and emotional problems variables ranged from 15.3 to 15.9%, apart from harsh parenting at age 6 years, which had a higher rate of missingness (33.8%). There were no differences between complete cases and those with missing data in harsh parenting at ages 6 and 11 years. However, compared to complete cases, those with missing data showed higher conduct problems at age 6 years, and lower levels of conduct and emotional problems at age 11 years. For the covariates, maternal education and maternal skin color had small amounts of missing data (< 2%), whereas maternal depression showed a higher rate of missingness (8.4%). Compared to complete cases, those with missing data at ages 6 and/or 11 years on either the CTSPC and/or SDQ reported higher incomes. Even for variables where there was a significant difference between those with versus without data, the respective effect sizes for the comparisons were small (*d*s ranging from − 0.09 to 0.13) (see Online Resource 1 for all pairwise comparisons).

We addressed missing data for all CTSPC and SDQ variables using multiple imputation. In Mplus, multiple imputation uses Bayesian analysis based on the Markov Chain Monte Carlo method, which simulates random draws from the posterior distribution of the missing scores [29, 30]. These scores were generated under the missing at random data loss mechanism, using all harsh parenting and child conduct and emotional problems variables, in addition to all covariates [29]. As we were interested in examining moderation effects by sex, we imputed data separately for boys and girls, which has been shown to preserve the multiple group data structure [31]. We used 40 imputed datasets, which has been shown to improve power, even for larger amounts of missing data [32]. The resulting analysis sample consisted of 3718 participants. Subsequently, we ran all models again using listwise deletion, resulting in a sample size of 2447 participants. The results were largely identical to the model based on multiple imputation (i.e., path coefficients were of similar magnitude; see Online Resource 2 for all model estimates based on listwise deletion). Due to moderate positive skew on all CTSPC and SDQ variables, all models were estimated using the Mplus MLR estimator, which produces standard errors which are robust to non-normality [33].

To evaluate the direction of associations between harsh parenting and child conduct and emotional problems, we assessed the importance of the parent-to-child and child-to-parent paths based on the strength of associations in the reciprocal models for the total sample. Wald’s test was used for determining whether path coefficients differed between boys and girls. All models were adjusted for maternal depression, smoking, alcohol consumption, relationship status, income, education, and skin color. Model estimates and the correlation matrix are based on imputed data and descriptive statistics on complete cases. Multiple imputation and path models were performed in Mplus, Version 8.1 [30]. All other data analyses were performed in RStudio, Version 1.1.447 [34].

## Results

### Descriptive statistics

At age 6 years, 14.7% (16.1% boys; 13.1% girls) of the sample showed high levels of conduct problems (i.e., a score of 4 or above) and 13.5% (13.0% boys; 14.0% girls) showed high levels of emotional problems (i.e., a score of 5 or above). At age 11 years, 13.0% (14.3% boys; 11.6% girls) and 20.0% (19.1% boys; 21.0% girls) of the sample showed high levels of conduct and emotional problems, respectively.

Compared to boys, girls were exposed to lower levels of harsh parenting and showed lower levels of conduct problems at ages 6 and 11 years, although effect sizes were small (*d*s ranging between − 0.10 and − 0.16, all *p*s < 0.01). No sex differences were observed for emotional problems at ages 6 and 11 years (see Table 1 for all pairwise comparisons).

**Table 1:** Descriptive statistics for the total sample and separated by sex

Variables (ranges in parentheses)	Total	Male	Female	Gender comparison	Effect size
	Mean (SD) or %	Mean (SD) or %	Mean (SD) or %	*t* (*df*) or *χ*^2^ (*df*)	*d* (95% CI) or OR (95% CI)
Harsh parenting (0–28)					*d*
Age 6	6.74 (4.26)	7.03 (4.35)	6.42 (4.13)	*t*(2737.8) = 3.81, *p* < 0.001	− 0.14 (− 0.22 to − 0.07)
Age 11	6.52 (4.49)	6.87 (4.64)	6.16 (4.29)	*t*(3484.5) = 4.71, *p* < 0.001	− 0.16 (− 0.23 to − 0.09)
Conduct problems (0–10)					
Age 6	1.53 (1.82)	1.65 (1.87)	1.40 (1.76)	*t*(3505.1) = 3.97, *p* < 0.001	− 0.14 (− 0.20 to − 0.07)
Age 11	1.39 (1.84)	1.48 (1.89)	1.29 (1.78)	*t*(3487.9) = 3.11, *p* = 0.002	− 0.10 (− 0.17 to − 0.04)
Emotional problems (0–10)					
Age 6	2.20 (2.05)	2.16 (2.02)	2.25 (2.09)	*t*(3459.2) = − 1.32, *p* = 0.19	0.04 (− 0.02 to 0.11)
Age 11	2.69 (2.33)	2.64 (2.34)	2.74 (2.33)	*t*(3475.2) = − 1.20, *p* = 0.23	0.04 (− 0.02 to 0.11)
Covariates					
Continuous					
Weekly family income (BRL)	200.87 (277.10)	205.13 (295.22)	196.24 (256.04)	*t*(4126.9) = 1.04, *p* = 0.30	− 0.03 (− 0.09 to 0.03)
Maternal education (years)	8.11 (3.47)	8.19 (3.49)	8.02 (3.45)	*t*(4079.4) = 1.58, *p* = 0.11	− 0.05 (− 0.11 to 0.01)
Maternal depression (0–30)	7.21 (5.04)	7.23 (5.00)	7.19 (5.08)	*t*(3765/3) = 0.25, *p* = 0.80	− 0.01 (− 0.07 to 0.06)
Binary					OR
Maternal prenatal smoking (yes)	27.6	27.2	27.9	*χ*^2^(1) = 0.29, *p* = 0.59	1.04 (0.90–1.19)
Maternal prenatal alcohol consumption (yes)	3.4	3.6	3.1	*χ*^2^(1) = 0.81, *p* = 0.37	0.86 (0.60–1.22)
Maternal relationship status (single)	16.3	17.1	15.5	*χ*^2^(1) = 1.82, *p* = 0.18	0.89 (0.75–1.06)
Maternal skin color (black/mixed race)	37.9	37.8	38.9	*χ*^2^(1) = 0.60, *p* = 0.44	1.05 (0.92–1.19)

Observed, rather than imputed values are presented.

BRL = Brazilian real (2.89 BRL = 1 USD in January 2004 when recruitment of the families commenced); CI = Confidence interval; *d* = Cohen′s *d*; *df* = degrees of freedom; OR = odds ratio; SD = Standard deviation

**Fig. 2:**
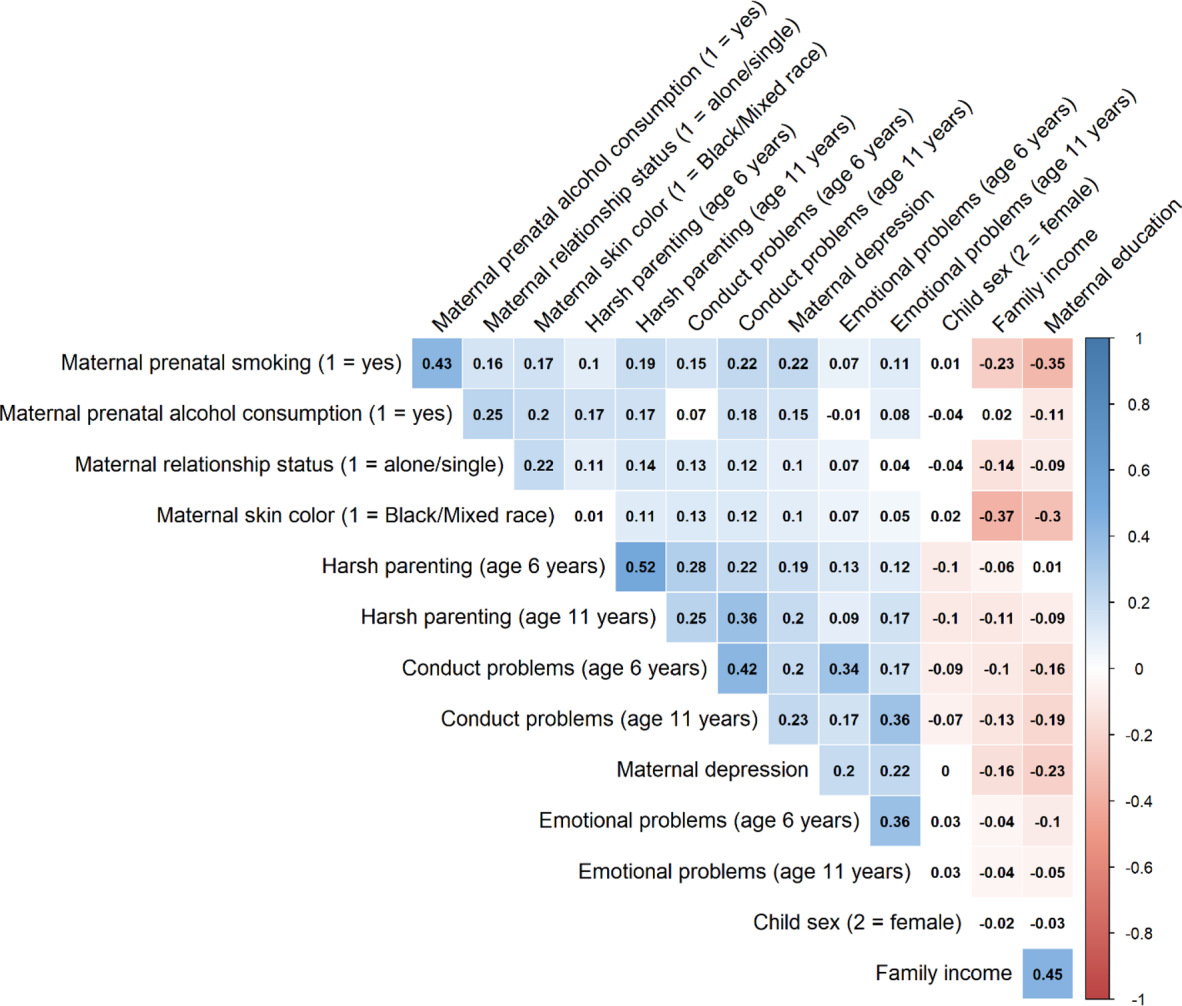
Correlation matrix of all variables used in the cross-lagged models. Imputed, rather than observed, values are presented. The color bar represents correlation coefficients from − 1 (red) to + 1 (blue). Blue squares represent significant positive correlations. Red squares represent significant negative correlations. Darker color tones represent larger correlation coefficients. White squares represent non-significant correlation coefficients at *p* < 0.05

Figure 2 shows the correlation matrix for all variables in the study. Harsh parenting, conduct problems, and emotional problems were moderately correlated both within and between time points (*r*s ranging between 0.09 and 0.52, all *p*s < 0.001). Harsh parenting showed higher concurrent and longitudinal associations with conduct problems (*r*s ranging between 0.22 and 0.37, all *p*s < 0.001) than emotional problems (*r*s ranging between 0.09 and 0.17, all *p*s < 0.001).

### Harsh parenting and child conduct problems

Table 2 shows standardized coefficients from path models of the relationships between harsh parenting and child conduct problems (unstandardized path coefficients are presented in Online Resource 3). There was a moderate degree of stability from ages 6 to 11 years for both harsh parenting and child conduct problems for the total sample, as well as for males and females separately, as indicated by significant autoregressive effects (*β*s ranging between 0.32 and 0.48, all *p*s < 0.001). In addition, a significant proportion of change over time in each variable was explained by temporally preceding parent-to-child and child-to-parent effects. For the total sample, as well as for males and females separately, harsh parenting at age 6 years predicted conduct problems at age 11, even after controlling for prior levels of conduct problems (*β*s ranging between 0.07 and 0.12, all *p*s < 0.01). Similarly, conduct problems at age 6 years predicted harsh parenting at age 11, even after controlling for prior levels of harsh parenting and independently of sex (*β*s ranging between 0.06 and 0.09, all *p*s < 0.05). There were no significant sex differences in autoregressive effects for harsh parenting (*χ*(1) = 0.590, *p* = 0.44) or conduct problems (*χ*(1) = 1.508, *p* = 0.22), indicating that the degree of stability over time did not differ between boys and girls. Similarly, there were no significant sex differences in cross-lagged effects from harsh parenting at age 6 years to conduct problems at age 11 (*χ*(1) = 1.528, *p* = 0.22), and from conduct problems at age 6 years to harsh parenting at age 11 (*χ*(1) = 0.346, *p* = 0.56), suggesting that the magnitude of parent- and child-effects did not differ between boys and girls.

**Table 2:** Path estimates using multiple imputation for the total sample and separated by sex

	Total sample (*N* = 3718)		Males (*N* = 1931)		Females (*N* = 1787)	
	*β* (SE)	*P*	*β* (SE)	*P*	*β* (SE)	*P*
Harsh parenting and conduct problems						
Autoregressive effects						
Conduct problems (age 6) → conduct problems (age 11)	0.351 (0.020)	< 0.001	0.374 (0.028)	< 0.001	0.321 (0.027)	< 0.001
Harsh parenting (age 6) → harsh parenting (age 11)	0.471 (0.016)	< 0.001	0.476 (0.022)	< 0.001	0.462 (0.024)	< 0.001
Cross-lagged effects						
Conduct problems (age 6) → harsh parenting (age 11)	0.076 (0.017)	< 0.001	0.063 (0.025)	= 0.010	0.085 (0.026)	= 0.001
Harsh parenting (age 6) → conduct problems (age 11)	0.093 (0.019)	< 0.001	0.073 (0.026)	= 0.005	0.117 (0.026)	< 0.001
Harsh parenting and emotional problems						
Autoregressive effects						
Emotional problems (age 6) → emotional problems (age 11)	0.332 (0.017)	< 0.001	0.326 (0.024)	< 0.001	0.335 (0.025)	< 0.001
Harsh parenting (age 6) → harsh parenting (age 11)	0.490 (0.015)	< 0.001	0.493 (0.021)	< 0.001	0.481 (0.023)	< 0.001
Cross-lagged effects						
Emotional problems (age 6) → harsh parenting (age 11)	0.003 (0.016)	= 0.859	0.004 (0.023)	= 0.857	0.004 (0.023)	= 0.865
Harsh parenting (age 6) → emotional problems (age 11)	0.043 (0.019)	= 0.026	0.027 (0.027)	= 0.315	0.066 (0.028)	= 0.017

All models were adjusted for maternal depression, smoking, alcohol consumption, relationship status, income, education, and skin color

*β* = standardized regression coefficient; SE = standard error; *P* = *p*-value

### Harsh parenting and child emotional problems

Table 2 shows standardized coefficients from path models of harsh parenting and child emotional problems (unstandardized path coefficients are presented in Online Resource 3). Similar to the model examining relationships between harsh parenting and conduct problems, there was a moderate degree of stability over time for both harsh parenting and emotional problems for the total sample, as well as for each sex separately, as indicated by significant autoregressive effects (*β*s ranging between 0.33 and 0.49, all *p*s < 0.001). However, in contrast to the reciprocal relationship between harsh parenting and child conduct problems, only temporally preceding parent-to-child, but not child-to-parent, effects predicted to change over time. More specifically, harsh parenting at age 6 years predicted emotional problems at age 11, even after controlling for prior levels of emotional problems (*β* = 0.04, *p* = 0.03). In contrast, emotional problems at age 6 years were not predictive of harsh parenting at age 11, after adjusting for prior levels of harsh parenting (*β* = 0.00, *p* = 0.86) and independently of sex. The observed parent-to-child effect was significant in females (*β* = 0.07, *p* = 0.02), but not in males (*β* = 0.03, *p* = 0.32). However, when directly comparing boys and girls, no significant sex differences were found (*χ*(1) = 1.098, *p* = 0.30). In addition, there were no sex differences in autoregressive effects for harsh parenting (*χ*(1) = 0.539, *p* = 0.46) or emotional problems (*χ*(1) = 0.040, *p* = 0.84), suggesting similar degrees of stability over time in boys and girls.

## Discussion

To our knowledge, this is the first study to use a prospective longitudinal design and a population-based sample to examine cross-lagged associations between harsh parenting and child conduct and emotional problems in a low- and middle-income country (LMIC). We found bidirectional effects between harsh parenting and child conduct problems (i.e., harsh parenting at age 6 years predicted conduct problems at age 11, even after adjusting for initial levels of conduct problems, and vice versa), but only a unidirectional relationship between harsh parenting and child emotional problems (i.e., harsh parenting at age 6 years predicted emotional problems at age 11, even after adjusting for baseline emotional problems, but not vice versa). We also examined whether sex moderated the strength or nature of the cross-lagged and autoregressive effects, but found no robust evidence for sex differences in these associations.

Previous studies have indicated that the effects of harsh parenting on child externalizing and internalizing problems may depend partly on cultural norms related to harsh parenting practices [12], suggesting heterogeneous effects across different cultural contexts. However, our findings from Brazil are in line with two meta-analyses of cross-lagged associations that almost exclusively included studies from HICs, which showed bidirectional effects for externalizing problems and unidirectional effects for internalizing problems [7, 8]. Effect sizes were small but in line with those reported in previous meta-analyses [7, 8]. On the basis of small effect sizes for parenting effects, some researchers have argued that there is insufficient evidence to categorically oppose physical punishment [35]. Others, however, have disputed this idea, stating the lack of evidence in support of physical punishment [36]. Some researchers have argued for a continuum of violence against children [37], with spanking and physical abuse both involving expression of harsh parenting and negative child outcomes, just to different degrees [38]. Thus, it should be noted that harsh parenting in the current study may better be described as harsh, aggressive, and abusive parenting. Nevertheless, there are substantial differences between countries in the prevalence of harsh parenting, and future research across cultural contexts is warranted [15].

The present findings provide support for transactional models between negative parental discipline and child conduct problems. According to Patterson’s coercive processes model of antisocial behavior [3, 4], dysfunctional parent–child interactions in early development lead to an incremental decline in the quality of the parent–child relationship. These coercive cycles may continue into middle and late childhood as well as adolescence and extend beyond the family context to affect behavior in school or within the peer group. According to social information processing theory and social learning theory [39, 40], children may internalize their parents’ harsh and abusive behavior, and, as a consequence, are unable to generate appropriate responses to situations of conflict and distress. Consequently, harsh parenting may play an important role in initiating child conduct problems. However, as Patterson notes, child characteristics, including, for example, difficult temperament, may negatively impact parenting practices [4].

The findings also provide evidence for a unidirectional parent-effects model of the association between negative parental discipline and child internalizing problems. Serbin et al. [41] found a negative feedback loop between parenting and child internalizing outcomes measured in the context of a longitudinal design, i.e., child internalizing problems at wave 1 led to an increase in positive parenting behaviors at wave 2, which, in turn, led to a decrease in internalizing problems at wave 3. In contrast, a recent meta-analysis found child internalizing symptoms led to reduced parental warmth and authoritative parenting, and increases in psychologically controlling and permissive parenting behaviors [8]. This implies that similar vicious cycles to those proposed by Patterson [3, 4] may apply to child internalizing problems, but with different expressions of ineffective parenting strategies. For example, cold, unsupportive, and neglectful parenting may lead to an increase in child internalizing problems and, similarly, a withdrawn child may evoke less parental engagement and fewer stimulating interactions. However, the current study was not designed to examine whether such effects exist in our sample.

In line with previous research, we found higher levels of conduct problems in boys compared to girls [42]. Furthermore, boys were exposed to higher levels of harsh parenting than girls, which may have contributed to them developing higher rates of conduct problems, and vice versa. However, despite these sex differences, the reciprocal relationship between harsh parenting and child conduct problems did not differ by sex. In contrast, the association between harsh parenting and child emotional problems was only significant for girls, but not boys. However, when we directly compared boys and girls, there was no significant sex difference in the strength of this effect. Unlike in previous studies, we did not find higher levels of emotional problems in girls compared to boys, which may partly explain the non-significant sex difference [43]. As studies on sex differences in these relationships have mostly been limited to HICs, with just two small-scale studies testing for sex differences in cross-lagged associations between harsh parenting and child externalizing problems in LMICs [44, 45], further research on this topic is needed in LMICs.

Key strengths of the current study include the use of a large, birth cohort sample from Brazil, with very high retention rates, and the availability of prospective longitudinal data. Furthermore, the majority of studies testing for cross-lagged associations between parenting dimensions and child externalizing and internalizing problems have been conducted in HICs [7, 8, 10]. Thus, we were able to examine whether the findings obtained in HICs extend to LMICs, including the direction of effects in the parent–child relationship and sex differences.

However, our study also had a number of limitations which should be considered when interpreting the findings: First, all measures were completed by a single rater, usually the mother, and therefore may have been subject to shared rater bias, which may have inflated associations between variables. Second, parents may under-report child emotional problems [46], especially in the case of girls [47]. Thus, future studies should attempt to mitigate against these issues by using both parent- and self-reports of child psychopathology. Third, there was selective attrition over time. These effects, however, were small and addressed through the use of multiple imputation, using an adequate number of imputed data sets, and taking the child’s sex into account [31, 32]. Furthermore, the findings were similar when listwise deletion was used to deal with missing data rather than multiple imputation. Fourth, the SDQ subscales showed modest internal consistency. Although, the SDQ is a widely used measure, the current results require replication, using a measure of child conduct and emotional problems with better psychometric properties. Fifth, with data available from only two time points, we were not able to examine a sequence of change (i.e., a feedback loop) between harsh parenting and child conduct and emotional problems, respectively, which would require data from a minimum of three time points. Data collection for the age 15-time point is currently underway, which will allow researchers to investigate these issues, in addition to modelling developmental trajectories of child conduct and emotional problems, respectively. Finally, the relationship between harsh parenting and child conduct problems may be in part explained by genetically mediated child-effects, especially in the case of less severe forms of harsh and abusive parenting [48]. However, the current study was not designed to investigate this possibility.

Given the bidirectional effects between harsh parenting and child conduct problems reported here and in other studies conducted in HICs [7], future interventions aimed at targeting harsh and abusive parenting to reduce conduct problems should also include child-focused components, directly targeting child behavior problems. In contrast, preventive interventions to address child internalizing problems may primarily focus on parent-focused components. In HICs, there is strong evidence for the effectiveness of parent training programs for child conduct problems, focusing on reducing harsh parenting and promoting positive parenting [49], with only preliminary evidence available from LMICs [50]. Upcoming trials will further elucidate the effectiveness of such parenting programs in Brazil [51].

In conclusion, we found reciprocal relationships between harsh parenting and child conduct problems, and unidirectional effects of harsh parenting on child emotional problems, with no significant sex differences observed in either model. Our findings highlight the detrimental impact of harsh parenting on child psychopathology and demonstrate the importance of targeting both parent- and child-effects in preventive interventions aiming to reduce harsh parenting and promote positive parenting.

## Supplementary Information

Below is the link to the electronic supplementary material.

Supplementary file1 (DOCX 18 KB)

Supplementary file2 (DOCX 16 KB)

Supplementary file3 (DOCX 16 KB)
