# Supplementary material for: Harsh parenting and child conduct and emotional problems: parent- and child-effects in the 2004 Pelotas Birth Cohort
Source: Eur Child Adolesc Psychiatry. 2021 Mar 18;31(8):1–11. doi: 10.1007/s00787-021-01759-w (PMC9343272; doi:10.1007/s00787-021-01759-w)
Supplement: Supplementary file 1 — Supplementary file1 (DOCX 18 KB) [file 787_2021_1759_MOESM1_ESM.docx]

**Online Resource 1** Attrition between complete cases (n = 2576) and those with at least one missing score (n = 1569) for the harsh parenting and/or child conduct and emotional problems variables

| **Variables** (ranges in parentheses) | **Complete cases** | **Missing** | **Comparison** | **Effect size** |
| --- | --- | --- | --- | --- |
|  | Mean (SD) or % | Mean (SD) or % | *t*(df) or *χ^2^*(df) | *d* (95% CI) or  OR (95% CI) |
| **Harsh parenting** (0-28) |  |  |  | *d* |
| Age 6 | 6.75 (4.26) | 6.50 (4.19) | *t*(188.98) = -0.75, *p* = .46 | -0.06 (-0.22 to 0.10) |
| Age 11 | 6.50 (4.45) | 6.59 (4.60) | *t*(1552.5) = 0.54, *p* = .59 | 0.02 (-0.06 to 0.10) |
| **Conduct problems** (0-10) |  |  |  |  |
| Age 6 | 1.47 (1.78) | 1.71 (1.92) | *t*(1551.5) = 3.38, *p* < .001 | 0.13 (0.06 to 0.21) |
| Age 11 | 1.43 (1.87) | 1.27 (1.73) | *t*(1722.5) = -2.26, *p* = .02 | -0.09 (-0.16 to -0.01) |
| **Emotional problems** (0-10) |  |  |  |  |
| Age 6 | 2.18 (2.04) | 2.27 (2.08) | *t*(1619) = 1.13, *p* = .26 | 0.05 (-0.02 to 0.13) |
| Age 11 | 2.74 (2.36) | 2.53 (2.25) | *t*(1674.5) = -2.41, *p* = .02 | -0.09 (-0.17 to -0.02) |
| **Covariates** |  |  |  |  |
| *Continuous* |  |  |  |  |
| Weekly family income (BRL) | 191.2 (257.56) | 216.74 (305.92) | *t*(2886.6) = 2.76, *p* = .006 | 0.09 (0.03 to 0.12) |
| Maternal education (years) | 8.03 (3.39) | 8.24 (3.60) | *t*(3125.2) = 1.88, *p* = .06 | 0.06 (-0.00 to 0.12) |
| Maternal depression (0-30) | 7.27 (5.07) | 7.09 (4.99) | *t*(2655.2) = -1.08, *p* = .28 | -0.04 (-0.10 to 0.03) |
| *Binary* |  |  |  | OR |
| Maternal prenatal smoking (yes) | 26.7 | 28.9 | *χ^2^*(1) = 2.42, *p* = .12 | 0.89 (0.78-1.03) |
| Maternal prenatal alcohol consumption (yes) | 3.2 | 3.7 | *χ^2^*(1) = 0.79, *p* = .37 | 0.86 (0.60-1.23) |
| Maternal relationship status (alone/single) | 16.0 | 16.8 | *χ^2^*(1) = 0.45, *p* = .50 | 0.94 (0.79-1.12) |
| Maternal skin color (Black/Mixed race) | 38.4 | 38.2 | *χ^2^*(1) = 0.02, *p* = .88 | 1.01 (0.89-1.15) |
| Child’s sex (female) | 48.3 | 47.6 | *χ^2^*(1) = 0.16, *p* = .69 | 1.03 (0.90-1.17) |

Note. Observed, rather than imputed values are presented. BRL = Brazilian real (1 USD in January 2004 when recruitment of the families commenced); CI = Confidence interval; d = Cohen’s d; df = Degrees of freedom; OR = Odds ratio; SD = Standard deviation.
