# Supplementary material for: Harsh parenting and child conduct and emotional problems: parent- and child-effects in the 2004 Pelotas Birth Cohort
Source: Eur Child Adolesc Psychiatry. 2021 Mar 18;31(8):1–11. doi: 10.1007/s00787-021-01759-w (PMC9343272; doi:10.1007/s00787-021-01759-w)
Supplement: Supplementary file 2 — Supplementary file2 (DOCX 16 KB) [file 787_2021_1759_MOESM2_ESM.docx]

**Online resource 2** Path estimates using *listwise deletion* for the total sample and separated by sex

|  | **Total sample** (*N* = 2447) | | | **Males** (*N* = 1261) | | | **Females** (*N* = 1186) | | |
| --- | --- | --- | --- | --- | --- | --- | --- | --- | --- |
|  | *B* (SE) | *β* (SE) | *P* | *B* (SE) | *β* (SE) | *P* | *B* (SE) | *β* (SE) | *P* |
| **Harsh parenting and conduct problems** | | | | | | | | | |
| *Autoregressive effects* |  |  |  |  |  |  |  |  |  |
| Conduct problems (age 6) →  conduct problems (age 11) | 0.350 (0.025) | 0.333 (0.023) | < .001 | 0.379 (0.035) | 0.367 (0.032) | < .001 | 0.312 (0.037) | 0.291 (0.033) | < .001 |
| Harsh parenting (age 6) →  harsh parenting (age 11) | 0.486 (0.020) | 0.469 (0.018) | < .001 | 0.502 (0.029) | 0.476 (0.026) | < .001 | 0.464 (0.027) | 0.458 (0.027) | < .001 |
| *Cross-lagged effects* |  |  |  |  |  |  |  |  |  |
| Conduct problems (age 6) →  harsh parenting (age 11) | 0.148  (0.050) | 0.059 (0.020) | = .003 | 0.118 (0.071) | 0.047 (0.028) | = .098 | 0.161 (0.068) | 0.064 (0.027) | = .018 |
| Harsh parenting (age 6) →  conduct problems (age 11) | 0.042 (0.009) | 0.097 (0.020) | < .001 | 0.034 (0.012) | 0.079 (0.028) | = .005 | 0.051 (0.013) | 0.117 (0.029) | < .001 |
| **Harsh parenting and emotional problems** | | | | | | | | | |
| *Autoregressive effects* |  |  |  |  |  |  |  |  |  |
| Emotional problems (age 6) →  emotional problems (age 11) | 0.396 (0.024) | 0.338 (0.020) | < .001 | 0.385 (0.029) | 0.326 (0.024) | < .001 | 0.376 (0.029) | 0.335 (0.025) | < .001 |
| Harsh parenting (age 6) →  harsh parenting (age 11) | 0.501 (0.019) | 0.483 (0.018) | < .001 | 0.524 (0.025) | 0.493 (0.021) | < .001 | 0.498 (0.025) | 0.481 (0.023) | < .001 |
| *Cross-lagged effects* |  |  |  |  |  |  |  |  |  |
| Emotional problems (age 6) →  harsh parenting (age 11) | 0.009 (0.038) | 0.004 (0.017) | = .809 | 0.010 (0.053) | 0.004 (0.023) | = .857 | 0.008 (0.047) | 0.004 (0.023) | = .865 |
| Harsh parenting (age 6) →  emotional problems (age 11) | 0.025 (0.011) | 0.046 (0.019) | = .019 | 0.015 (0.015) | 0.027 (0.027) | = .315 | 0.037 (0.016) | 0.066 (0.028) | = .039 |

***Note.*** All models were adjusted for maternal depression, smoking, alcohol consumption, relationship status, income, education, and skin color.
*B* = unstandardized regression coefficient; *β* = standardized regression coefficient; SE = standard error; *P* = *p*-value.
