# Supplementary material for: Harsh parenting and child conduct and emotional problems: parent- and child-effects in the 2004 Pelotas Birth Cohort
Source: Eur Child Adolesc Psychiatry. 2021 Mar 18;31(8):1–11. doi: 10.1007/s00787-021-01759-w (PMC9343272; doi:10.1007/s00787-021-01759-w)
Supplement: Supplementary file 3 — Supplementary file3 (DOCX 16 KB) [file 787_2021_1759_MOESM3_ESM.docx]

| **Online resource 3** Unstandardized regression coefficients using multiple imputation for the total sample and separated by sex | | | | | | |
| --- | --- | --- | --- | --- | --- | --- |
|  | **Total sample** (*N* = 3718) | | **Males** (*N* = 1931) | | **Females** (*N* = 1787) | |
|  | *B* (SE) | *P* | *B* (SE) | *P* | *B* (SE) | *P* |
| **Harsh parenting and conduct problems** | | | | | | |
| *Autoregressive effects* |  |  |  |  |  |  |
| Conduct problems (age 6) →  conduct problems (age 11) | 0.354 (0.021) | < .001 | 0.376 (0.030) | < .001 | 0.326 (0.029) | < .001 |
| Harsh parenting (age 6) →  harsh parenting (age 11) | 0.494 (0.018) | < .001 | 0.506 (0.026) | < .001 | 0.478 (0.025) | < .001 |
| *Cross-lagged effects* |  |  |  |  |  |  |
| Conduct problems (age 6) →  harsh parenting (age 11) | 0.189 (0.044) | < .001 | 0.159 (0.062) | = .010 | 0.211 (0.063) | = .001 |
| Harsh parenting (age 6) →  conduct problems (age 11) | 0.040 (0.008) | < .001 | 0.031 (0.011) | = .005 | 0.050 (0.011) | < .001 |
| **Harsh parenting and emotional problems** | | | | | | |
| *Autoregressive effects* |  |  |  |  |  |  |
| Emotional problems (age 6) →  emotional problems (age 11) | 0.382 (0.020) | < .001 | 0.385 (0.029) | < .001 | 0.376 (0.029) | < .001 |
| Harsh parenting (age 6) →  harsh parenting (age 11) | 0.514 (0.017) | < .001 | 0.524 (0.025) | < .001 | 0.498 (0.025) | < .001 |
| *Cross-lagged effects* |  |  |  |  |  |  |
| Emotional problems (age 6) →  harsh parenting (age 11) | 0.006 (0.035) | = .859 | 0.010 (0.053) | = .857 | 0.008 (0.047) | = .865 |
| Harsh parenting (age 6) →  emotional problems (age 11) | 0.023 (0.011) | = .026 | 0.015 (0.015) | = .315 | 0.037 (0.016) | = .017 |
| ***Note.*** All models were adjusted for maternal depression, smoking, alcohol consumption, relationship status, income, education, and skin color. *B* = unstandardized regression coefficient; SE = standard error; *P* = *p*-value. | | | | | | |
